# Tks5-dependent formation of circumferential podosomes mediates cell-cell fusion

**DOI:** 10.1186/ar3690

**Published:** 2012-02-09

**Authors:** Tsukasa Oikawa, Masaaki Oyama, Hiroko Kozuka-Hata, Shunsuke Uehara, Nobuyuki Udagawa, Hideyuki Saya, Koichi Matsuo

**Affiliations:** 1 Laboratory of Cell and Tissue Biology, Institute for Integral Medical Research, School of Medicine, Keio University, Shinanomachi 35, Shinjuku-ku, Tokyo 160-8582, Japan; 2 Medical Proteomics Laboratory, Institute of Medical Science, University of Tokyo, 4-6-1 Shirokanedai, Minato-ku, Tokyo 108-8639, Japan; 3 Department of Biochemistry, Matsumoto Dental University, 1780 Gobara, Hiro-oka, Shiojiri, Nagano 399-0781, Japan; 4 Division of Gene Regulation, Institute for Advanced Medical Research, School of Medicine, Keio University, Shinanomachi 35, Shinjuku-ku, Tokyo 160-8582, Japan; 5 CREST, Japan Science and Technology Agency, Tokyo, Japan

## 

Multinucleation of osteoclasts during osteoclastogenesis requires dynamic rearrangement of the plasma membrane and cytoskeleton, and this process involves numerous previously characterized factors. However, the mechanism underlying osteoclast fusion remains obscure. Live-imaging analysis of osteoclastogenesis revealed that the products of PI3-kinase are enriched at the sites of osteoclast fusion. Among the downstream molecules whose expression was screened, the expression of Tks5, an adaptor protein with the phox homology (PX) domain with multiple Src homology 3 domains, was induced during osteoclastogenesis. Tks5 was localized in the podosomes and fusing membranes of osteoclasts, and reducing its expression impaired both formation of circumferential podosomes and osteoclast fusion without altering osteoclast differentiation. In addition, the expression of a deletion mutant of the PX domain abrogated circumferential podosome formation as well as osteoclast fusion, suggesting that Tks5-dependent circumferential podosomes function as fusion machinery during osteoclastogenesis. As Tks5 is known to promote the formation of podosomes/invadopodia in transformed/cancer cells, we tested if these cells also have the potential to fuse with osteoclasts. Among the cells tested, B16F0 melanoma cells formed circumferential podosomes with Tks5 accumulation in the presence of RANKL, TGFβ and TNFα. Co-culture of B16F0 melanoma cells with osteoclasts in an inflammatory milieu promoted increased formation of melanoma-osteoclast hybrid cells. Our results revealed a previously unknown mechanism of regulation of both circumferential podosome formation and cell-cell fusion by Tks5.

